# Anti-Inflammatory Potential of the Oleoresin from the Amazonian Tree *Copaifera reticulata* with an Unusual Chemical Composition in Rats

**DOI:** 10.3390/vetsci8120320

**Published:** 2021-12-10

**Authors:** José Sousa de Almeida Júnior, Éden Bruno Sousa da Silva, Tânia Mara Pires Moraes, Aline Aparecida München Kasper, Adilson Sartoratto, Leopoldo Clemente Baratto, Elaine Cristina Pacheco de Oliveira, Euzebio Oliveira, Lauro Euclides Soares Barata, Antonio Humberto Hamad Minervino, Waldiney Pires Moraes

**Affiliations:** 1 Laboratório de Farmacologia Experimental, Universidade Federal do Oeste do Pará—UFOPA, Santarém 68040-255, Brazil; jsalmeidajr@hotmail.com (J.S.d.A.J.); edenbrunoss@gmail.com (É.B.S.d.S.); taniafarma@gmail.com (T.M.P.M.); waldineypires@gmail.com (W.P.M.); 2 Laboratório de Produção e Desenvolvimento de Produtos Naturais Bioativos (P&DBio), Universidade Federal do Oeste do Pará—UFOPA, Santarém 68040-255, Brazil; aliny_@msn.com (A.A.M.K.); lauroesbarata@gmail.com (L.E.S.B.); 3 Centro Pluridisciplinar de Pesquisas Químicas, Biológicas e Agrícolas, Universidade de Campinas—UNICAMP, Campinas 13083-970, Brazil; adilson@cpqba.unicamp.br; 4 Laboratório de Farmacognosia Aplicada, Universidade Federal do Rio de Janeiro—UFRJ, Rio de Janeiro 21941-901, Brazil; leopoldo.ufrj@gmail.com; 5 Laboratório de Biotecnologia Vegetal, Universidade Federal do Oeste do Pará—UFOPA, Santarém 68040-255, Brazil; ecp.oliveira@yahoo.com.br; 6 Laboratório de Neuroproteção e Neurorregeneração Experimental, Universidade Federal do Pará—UFPA, Belém 66075-110, Brazil; euzebio21@yahoo.com.br; 7 Laboratório de Sanidade Animal, LARSANA, Universidade Federal do Oeste do Pará—UFOPA, Santarém 68040-255, Brazil

**Keywords:** Fabaceae, *Copaifera reticulata*, β-Bisabolene, anti-inflammatory activity, inflammation, medicinal plants

## Abstract

*Copaifera reticulata* Ducke is a popularly known species known as copaíba that is widely spread throughout the Amazon region. The tree yields an oleoresin which is extensively used in local traditional medicine mainly as an anti-inflammatory and antinociceptive agent. The aim of the present study was to assess the anti-inflammatory potential of this oleoresin obtained from a national forest in the central Amazon which presented an unusual chemical composition. The chemical composition of volatile compounds of oleoresin was analyzed by gas chromatography-mass spectrometry. The acute toxicity assay was performed with a single dose of 2000 mg/kg. The anti-inflammatory potential was evaluated by carrageenan-induced paw edema and air pouch assays using four different *C. reticulata* oleoresin concentrations (10, 100, and 400 mg/kg). The exudate was evaluated for nitrite concentration through the colorimetric method and for TNF-α, IL-1β, and PGE_2_ by ELISA. *C. reticulata* oleoresin collected in the Amazonian summer contained six major sesquiterpene compounds (*β*-bisabolene, *cis*-eudesma-6,11-diene, *trans*-*α*-bergamotene, *β*-selinene, *α*-selinene, and *β*-elemene) and was nontoxic at a dose of 2000 mg/kg, showing low acute toxicity. Different from oleoresin obtained from other sites of the Brazilian Amazon, the major volatile compound found was β-Bisabolene with 25.15%. This β-Bisabolene-rich oleoresin reduced the formation of paw edema induced by carrageenan and reduced the global number of cells in the air pouch assay, as well as exudate volume and nitrite, TNF-α, IL-1β, and prostaglandin E_2_ levels (*p* < 0.05). *C. reticulata* oleoresin with a high β-Bisabolene concentration showed anti-inflammatory activity, reducing vascular permeability and consequently edema formation, and thus reducing cell migration and the production of inflammatory cytokine, confirming its traditional use by local Amazonian communities.

## 1. Introduction

Plants are a major primary source for molecules with a wide range of biological activity, from classical activities such as anti-inflammatory, and antimicrobial [1,2] to additional biological properties such as anticancer activity [3,4] and microbiome regulation [5], with recent studies showing that Amazon can be a source of plant with a broad array of biological activities [6,7].

Inflammation is a response of the immune system to tissue damage caused by biological, mechanical, or chemical stimuli [8] and is mediated by many signaling molecules released by local cells in the presence of an injury. These substances are responsible for the formation of the main signs in most cases, but in others, when some inflammatory mediators are dysregulated (for example, nitric oxide (NO), eicosanoids, and cytokines), extremely severe pathological disorders can emerge as a consequence, such as septic shock, autoimmune diseases and chronic inflammatory diseases [9].

In communities in the Amazon region, people use natural products in traditional medicine to treat some health problems, such as inflammation and pain [10,11]. *Copaifera reticulata* Ducke (Fabaceae) is a common Amazonian species known for its ethnopharmacological application. It is a tree popularly known as copaíba or pau d’óleo that grows to a height of up to 40 m [10] with a trunk rich in an oleoresin that is extracted by perforation of the wood. Oleoresin was first used in folk medicine as an anti-inflammatory and analgesic agent after native Indians observed the behavior of wounded animals, which rubbed their bodies against the trunk of copaíba trees in an attempt to heal their wounds. Indians also used to apply the oleoresin to the navels of newborns and to warriors who returned from their battles with exposed wounds [12].

The anti-inflammatory activity of *Copaifera* sp. oleoresin samples has been studied during the last decade after extraction from *C. cearensis* [13], *C. duckei* [14], and *C. multijuga* [13,15,16,17,18,19]. *C. reticulata* oleoresin has also been previously investigated for its anti-inflammatory activity, but only in the zymosan-induced pleurisy model in Swiss mice [6], in the tongue injury model in male Wistar rats [20], and in the arthritis model in male Holtzman rats [21].

Previous research in vitro using cell culture revealed anti-inflammatory activity of *Copaifera* species. Dias et al. [22] observed in *Copaifera*-treated cells a reduction in the production of tumor necrosis factor, interferon-gamma, interleukin (IL)-17, nitric oxide, and hydrogen peroxide. Destryana et al. [23] found that copaiba was able to reduce the levels of nitric oxide, IL-6, IL-8, and IL-1β produced by macrophages.

*C. reticulata* oleoresin contains sesquiterpenes in the volatile fraction and diterpenes in the resinous fraction. The major compounds reported are sesquiterpenes: the most common is *β*-caryophyllene, which has anti-inflammatory, antitumor, and antimicrobial activity, followed by *β*-bisabolene, which has anti-inflammatory and analgesic properties [24,25]. Diterpenes are minor compounds, but they have some important activities such as cytotoxicity against tumor cells (kolavenol and hardwickiic acid) and anti-inflammatory activities (copalic acid) [10].

Considering that the chemical components of oleoresin of *Copaifera reticulata* have significant seasonal influence on their constitution and that the anti-inflammatory pharmacological activity of oleoresin extracted from the copaibeiras in the western region of Pará, in the Tapajós National Forest (Belterra Para), has not yet been reported in in vivo or in vitro studies, the objective of the present study was to evaluate the anti-inflammatory effects of *C. reticulata* oleoresin using male Wistar rats and carrageenan-induced inflammation as an experimental model.

## 2. Material and Methods

### 2.1. Plant Material

*C. reticulata* oleoresin was collected in Floresta Nacional do Tapajós (FLONA) during the dry period (Amazonian summer) of 2011 at Belterra, Pará State, Brazil. The species was identified by the taxonomist Regina Célia Viana Martins da Silva and a voucher specimen was deposited in the Herbarium of Embrapa Oriental under registration NID: 69/2011. Oil extraction for scientific purposes was authorized and approved by the Brazilian regulatory agency (protocol number: 44380-1). The trees were mechanically perforated with a traditional auger (2 cm in diameter and 45 cm in length) producing two holes at 1.00 and 1.50 m from the soil, respectively. Oleoresin samples were collected and stored in plastic containers protected from light and then transferred to glass vials (10 mL) for later analysis. After the full flow of oleoresin, the tree holes were sealed with a PVC-type pipe 0.75 cm in diameter and 10 cm in length) covered with a plastic cap in order to facilitate the next collections and to avoid wood waste [26].

The oleoresin tested in this study was endotoxin free, as determined according to the instructions of the Lonza Kinetic-QCL Chromogenic Limulus Lysate (LAL) Endotoxin Assay Kit (Walkersville, MD, USA), with a sensitivity range of 0.005–50.0 EU/mL.

### 2.2. Chemical Characterization of Volatile Compounds from C. reticulata Oleoresin

The chemical composition of the volatile compounds from oleoresin was analyzed by gas chromatography-mass spectrometry (GC-MS) using an Agilent gas chromatographer model HP-6890, a selective mass detector Agilent model HP-5975, a capillary column HP-5 MS (30 m × 0.25 mm × 0.25 µm), injector temperature = 250 °C, column temperature = 80 °C, heating rate = 5 °C/min up to 280 °C (20 min) and detector temperature = 300 °C; carrier gas = helium (flow of 1 mL/min); selective mass detector operating at 70 eV, *m/z* = 30 to 500 a.m.u. Oleoresin was dissolved in ethyl acetate at 20 mg/mL. Major volatile compounds were identified by comparison between the retention indices (RI) of the investigated substances and the RI available in the NIST library.

### 2.3. Animals

Wistar rats (140–220 g) 8 to 12 weeks of age were used in the experiments. Animals were kept in individual plastic cages (supplied with wood sawdust changed every three days) at 23 ± 0.5 °C on a dark-light cycle of 12 h/12 h and had access to water and food ad libitum prior to and during the experiments. The experiments were approved by the Ethics Committee for Animal Use, Federal University of Western Pará-UFOPA (protocol number: 07004/2013) and were conducted according to best practices of animal welfare.

We used a total of 62 rats, 12 of which were female and used for the acute toxicity study. In the study of paw edema, 25 male rats were used and in the air pouch test another 25 male rats were used.

### 2.4. Drugs and Solutions

The following drugs and solutions were used to perform the experiments: ethanol (Synth PA-ACS, Diadema, Brazil), carrageenan (Sigma Chemical Co., St. Louis, MO, USA), sodium pentobarbital (Hypnol^®^ 3% Fontoveter, Itapira, Brazil), Tween 80 (Sigma Chemical Co.), dexamethasone disodium phosphate (Sigma Chemical Co.), and Griess reagent (Sigma Chemical Co.).

Oleoresin was diluted in 15% ethanol P.A. and 0.312% Tween 80. The vehicle consisted of 0.312% Tween 80, 15% ethanol, and 0.9% saline solution.

### 2.5. Acute Toxicity Assay

The acute toxicity assay followed the guidelines of the Organization for Economic Co-operation and Development (OCDE)-423/2001 [27]. This protocol states that, when previous studies have assessed the acute toxicity of a known sample, the initial dose used can be the maximum dose without side effects. As previously reported by Sachetti et al. [25], *C. reticulata* oleoresin was tested in a single dose of 2000 mg/kg. We used twelve 9-week-old female rats weighing 150 to 180 g. Six rats received the oleoresin and six were kept as the control group, receiving the same dose (2000 mg/kg) of water. The acute toxicity assay was performed with three rats per group (control and treated) and repeated after 14 days with another three rats per group, totaling twelve rats. Several parameters such as general activity, irritability, contortion, ataxia, tremors, convulsions, piloerection, hypothermia, breathing, cyanosis, hyperemia, and death were analyzed.

### 2.6. Experimental Design

The animals were treated with three different doses of *C. reticulata* oleoresin (low, medium, and high), which were defined according to the acute toxicity assay, corresponding to 1/200, 1/20, and 1/5 of the maximal dose with biological safety (2000 mg). The standard anti-inflammatory drug was dexamethasone (0.6 mg/kg, orally).

A total of 50 rats were submitted to two different anti-inflammatory activity tests: carrageenan-induced paw edema assay (*n* = 25) and carrageenan-induced air pouch assay (*n* = 25). The first group was treated only with vehicle and the pro-inflammatory stimulus (*Carrageenan)*. In the second group, the animals were treated with the standard drug and pro-inflammatory stimulus. The third, fourth, and fifth groups were treated with oleoresin at 10, 100, and 400 mg/kg, respectively, and the pro-inflammatory stimulus.

### 2.7. Anti-Inflammatory Activity

#### 2.7.1. Carrageenan-Induced Paw Edema Assay

Paw edema was measured as described by Koo et al. [28]. Animals (*n* = 5 per group) were treated orally with vehicle, dexamethasone (0.6 mg/kg), and oleoresin (10, 100, and 400 mg/kg,); 0.2 mL of 1% carrageenan was injected into the intraplantar region of the right hind paw and an equal volume of saline solution was injected into the left hind paw. The volume (mL) of the paw was measured with a digital plethysmometer immediately after carrageenan administration (0) and at later intervals (1, 2, 3, and 4 h) and the difference in volume between the two paws was calculated.

#### 2.7.2. Carrageenan-Induced Air Pouch Assay

Air pouches were produced in the animals by subcutaneous injection with 20 mL of sterile air in the intrascapular region of Wistar rats as described by Tao et al. [29]. Pouches were reinflated with 10 mL air after the 3rd and 6th days. Oral treatment (*n* = 5 per group) with vehicle, dexamethasone (0.6 mg/kg), and oleoresin (10, 100, and 400 mg/kg) was performed on the 9th day. One hour after the beginning of treatment, 2 mL of 1% carrageenan was administered directly into the pouch. On the 10th day, the animals were sacrificed with a sodium pentobarbital overdose 16 h after the stimulus with carrageenan. After incision in the wall of the air pouch, the content was collected with a sterile Pasteur pipette and the exudate volume was measured. Cells were counted (cells/mm^3^) in a Neubauer chamber and the cytokines listed below were determined.

Euthanasia was performed by intraperitoneal injection of a sodium pentobarbital overdose, corresponding to two to three times the dose considered anesthetic (100 mg/kg), as recommended by Guidelines for the Practice of Euthanasia of the National Council for the Control of Animal Experimentation (CONCEA) [30].

#### 2.7.3. Determination of Nitrite, Tumor Necrosis Factor-Alpha (TNF-α), Interleukin (IL-1β) and Prostaglandin E_2_ (PGE_2_) in the Exudate

Nitrite levels were measured in the exudate samples by the addition of 500 µL Griess reagent to 500 µL of exudate in a spectrophotometer at 540 nm. Nitrite concentration was determined by comparison with the calibration curve with serial dilutions of sodium nitrite as described by Koo et al. [19]. The concentrations of TNF-α, IL-1β, and PGE_2_ in the exudate were determined with ELISA kits for rats (eBioscience, San Diego, CA, USA) and the results are expressed as pg/mL

### 2.8. Statistical Analysis

Data were analyzed statistically using GraphPad Prism (6.0) software and submitted to analysis of variance (one-way or two-way ANOVA). The Newman–Keuls or Bonferroni multiple comparison test was used for comparison between groups. Results were considered significant for values of * *p* < 0.05; ** *p* < 0.01; *** *p* < 0.001.

## 3. Results

### 3.1. Chemical Composition of Volatile Compounds of C. reticulata Oleoresin

According to Table 1, the characterization of oleoresin volatile compounds showed the presence of six major sesquiterpenes corresponding to 74.8% of the total compounds: *β*-bisabolene (25.15%), *cis*-eudesma-6,11-diene (14.20%), *trans*-*α*-bergamotene (12.76%), *β*-selinene (8.70%), *α*-selinene (7.03%), and *β*-elemene (6.96%). It is important to note that the percentages of the compounds listed in Table 1 are related to the total number of compounds identified in the GC-MS analysis; they are not the percentage of the compounds in the extract and that the GC-MS analysis identified only volatile compounds.

Table 2 presents a comparative result from the chemical composition of volatile compounds from *C. reticulata* obtained from different areas of the Brazilian Amazon. Our samples were obtained from FLONA Tapajós, located in the central Amazon, and had different chemical compositions from samples from Belém within the same state (Pará) but located in the eastern Amazon and from samples obtained from Acre State located in the western Amazon. While the same plant from other sites from the Amazon had limited amounts of sesquiterpenes, the sample used in the present study yielded as much as 25% of one single compound (β-Bisabolene).

### 3.2. Acute Toxicity

The animals treated with a 2000 mg/kg dose of oleoresin did not show any signs of toxicity and no deaths occurred. For this reason, it was established that the acute oral toxicity of the oleoresin was higher than 2000 mg/kg and the product was classified as category 5 with a high level of biological safety.

### 3.3. Paw edema

**Figure 1:**
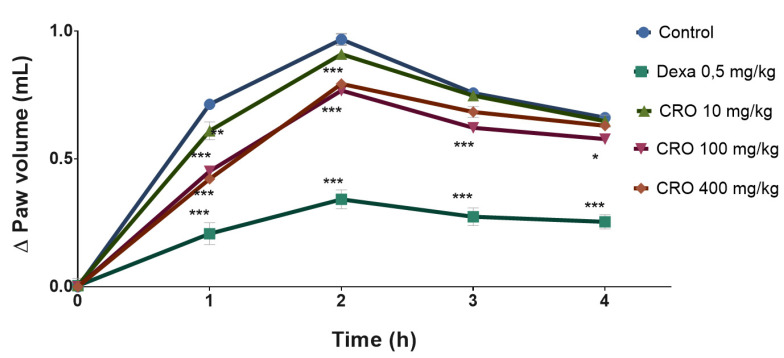
Effect of *C. reticulata* oleoresin (10, 100, and 400 mg/kg) and dexamethasone (0.6 mg/kg) administered orally to Wistar rats on the edematogenic stimulus induced by 1% carrageenan (200 µL, intraplantar). Each point represents the mean ± SE of a group of 5 animals. * *p* < 0.05; ** *p* < 0.01; *** *p* < 0.001 compared to control; two-way ANOVA, Bonferroni test.

The oleoresin was able to reduce edema in the first hour of evaluation by 14.56% (0.61 ± 0.08 mL), 36.69% (0.45 ± 0.02 mL) and 40.89% (0.42 ± 0.03 mL) at 10, 100, and 400 mg/kg, respectively, compared to the control (0.71 ± 0.01). In the second hour, only the groups treated with 100 and 400 mg/kg oleoresin showed a significant antiedema effect, with a reduction in paw edema of 20.66% (0.77 ± 0.02 mL) and 17.97% (0.79 ± 0.04 mL), respectively, compared to the vehicle-treated group (0.97 ± 0.05 mL). After the third (T3) and fourth (T4) hours, only the group treated with 100 mg/kg oleoresin showed a significant antiedema activity, with a reduction in edema of 17.94% (0.62 ± 0.04 mL) and 12.72% (0.58 ± 0.04 mL), respectively, compared to the vehicle-treated group (T3 = 0.75 ± 0.02 and T4 = 0.66 ± 0.03 mL). Dexamethasone, 0.6 mg/kg, prevented edema formation along the 4 h evaluation (Figure 1).

### 3.4. Volume of Exudate from the Air Pouch

**Figure 2:**
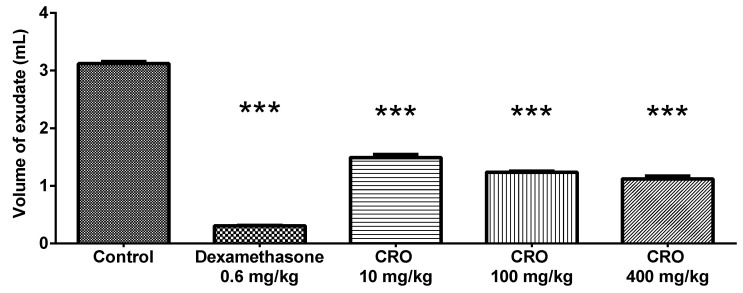
Volume of exudate produced in the air pouch. Effect of *C. reticulata* oleoresin—CRO (10, 100, and 400 mg/kg, orally) and dexamethasone (0.6 mg/kg, orally) on Wistar rats using the air pouch model. Each point represents the mean ± SE of a group of 5 animals. *** *p* < 0.001 compared to control (vehicle); one-way ANOVA, Newman–Keuls multiple comparison test.

Oleoresin showed a dose-dependent effect on the production of exudate in the air pouch, reducing it by 52.22% (1.49 ± 0.12 mL), 60.43% (1.23 ± 0.04 mL), and 64.08% (1.12 ± 0.01 mL) at 10, 100, and 400 mg/kg, respectively, compared to the total volume produced by the control group (3.12 ± 0.08 mL). Dexamethasone reduced the exudate volume by 90.25% (0.30 ± 0.02) (Figure 2).

### 3.5. Cell Count in the Air Pouch Exudate

**Figure 3:**
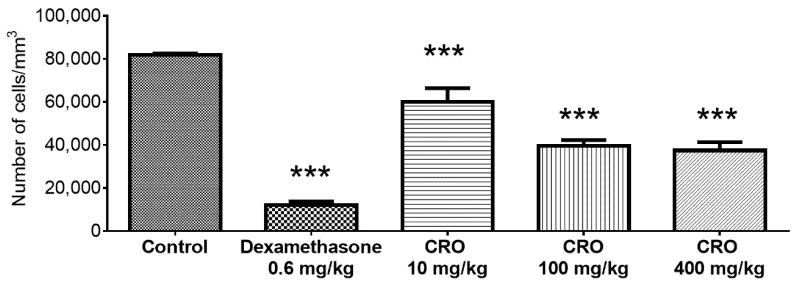
Count of the total number of cells of the exudate in the air pouch. Effect of *C. reticulata* oleoresin—CRO (10, 100, and 400 mg/kg, orally) and dexamethasone (0.6 mg/kg, orally) on Wistar rats using the air pouch model. Each point represents the mean ± SE of a group of 5 animals. *** *p* < 0.001 compared to control (vehicle); one-way ANOVA, Newman–Keuls multiple comparison test.

Oleoresin reduced the number of cells in the air pouch. Dexamethasone inhibited cell recruitment by 85.40% (11.98 ± 4.03 cells/mm^3^) compared to the control (81.92 ± 1.29 cells/mm^3^). The groups treated with 10, 100, and 400 mg/kg oleoresin showed a reduction in cell numbers of 26.80% (59.97 ± 14.34 cells/mm^3^), 51.60% (39.66 ± 5.84 cells/mm^3^), and 54.30% (37.45 ± 8.50 cells/mm^3^), respectively (Figure 3).

### 3.6. Determination of Nitrite Levels

**Figure 4:**
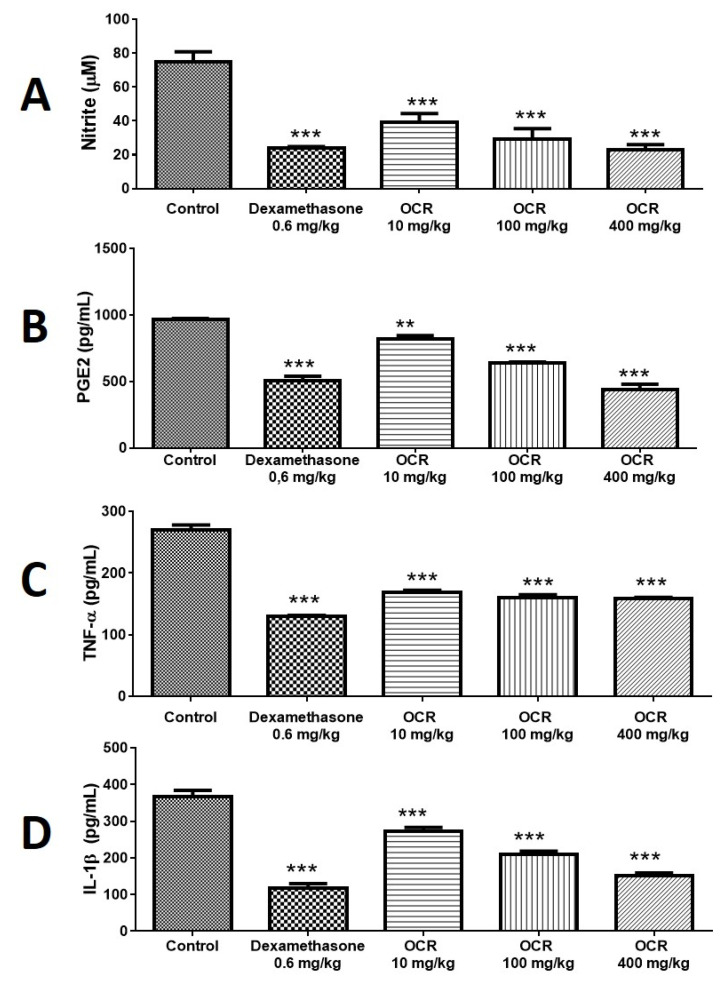
Effect of *C. reticulata* oleoresin—CRO (10, 100, and 400 mg/kg) and dexamethasone (Dexa) (0.6 mg/kg) administered orally to Wistar rats on inflammation mediators using the air pouch model: determination of nitrite (**A**), PGE_2_ (**B**), TNF-α (**C**), and IL-1β (**D**) in the air pouch exudate. Each point represents the mean ± SE of a group of 5 animals. ** *p* < 0.01; *** *p* < 0.001, compared to control (vehicle); one-way ANOVA, Newman–Keuls multiple comparison test.

Oleoresin (10, 100, and 400 mg/kg) significantly reduced nitrite levels in the air pouch by 44.00% (39.45 ± 2.78 µM), 56.90% (30.40 ± 1.20 µM), and 64.00% (25.35 ± 0.75 µM), respectively. Dexamethasone reduced these levels by 65.80% (24.19 ± 0.70 µM) (Figure 4A).

### 3.7. Determination of PGE_2_ Levels

Oleoresin promoted a dose-dependent response of PGE_2_ levels at concentrations of 10, 100, and 400 mg/kg, reducing the production of this prostaglandin by 15.11% (817.60 ± 27.54 pg/mL), 33.64% (639.20 ± 8.37 pg/mL), and 54.73% (436.00 ± 43.63 pg/mL), respectively, compared to the control (963.20 ± 10.28 pg/mL). Dexamethasone reduced the concentration of PGE_2_ by 47.78% (503.00 ± 35.83 pg/mL) (Figure 4B).

### 3.8. Determination of TNF-α Levels

Oleoresin, 10, 100, and 400 mg/kg, had a significant effect, reducing the TNF-α concentration by 37.26% (169.40 ± 2.71 pg/mL), 40.45% (160.80 ± 4.49 pg/mL), and 41.41% (158.20 ± 2.44 pg/mL), respectively, compared to the control (270.00 ± 7.84 pg/mL). Dexamethasone reduced TNF-α levels by 48.00% (129.60 ± 1.86 pg/mL) (Figure 4C).

### 3.9. Determination of IL-1β Levels

Oleoresin, 10, 100, and 400 mg/kg, significantly reduced IL-1β levels by 25.93% (272.00 ± 11.58), 42.86% (209.80 ± 8.44), and 58.93% (150.80 ± 8.07), respectively, compared to control (367.20 ± 17.49). Dexamethasone reduced IL-1β levels by 67.75% (118.40 ± 11.18 pg/mL) (Figure 4D).

## 4. Discussion

The chemical characterization of volatile compounds from *C. reticulata* oleoresin showed the presence of sesquiterpenes, including β-bisabolene (25.15%), eudesma cis-6,11-diene (14.20%), and *trans-α*-bergamotene (12.76%). These results are in agreement with Bardají et al. [31], who reported the presence of β-bisabolene (24.91%) and *trans-α*-bergamotene (21.99%) as the main oleoresin volatile compounds of *C. reticulata*, collected from Brasil Novo, in the state of Pará, Brazil. However, according to a previous report in Brazil [10], there is wide variability in the chemical composition of oleoresin of the genus Copaifera, depending on the species, location, seasonal variations, light intensity, soil nutrients, and attack by pathogens and herbivores. The chemical composition of volatile compounds of *C. reticulata* was found with a wide variability of the sesquiterpene fraction [32]. One limitation of the present study was the chemical analytical method, since GC-MS only identified volatile compounds and was based only on comparison of RI values to those provided by the NIST library. In addition, studies carried out by Veiga [13] and Gomes et al. [16] demonstrated the different chemical compositions and concentrations of compounds in the same species of *C. reticulata*. However, all samples of oleoresin of that species contained β-bisabolene. In a comparative analysis of the chemical composition of oleoresin from *Copaifera reticulata* obtained in the present study to previous reports [13,16], the presence of β-bisabolene was observed in the three oleoresins, even at different concentrations. However, the major compounds in the three different studies did not match, thus demonstrating variability in the chemical composition of the species. Therefore, although the anti-inflammatory activity of *C. reticulata* has already been reported, our study corroborates this result since the chemical composition of volatile compounds of the tested oleoresin, as well as the representativeness of the compounds, are also different from the oleoresin described in the literature.

Oleoresin was nontoxic at a dose of 2000 mg/kg, showing low acute toxicity. It significantly reduced the paw edema in the first hour in a dose-dependent manner during the phase of release of mediators such as histamine, serotonins, and/or kinins. By the second hour (second phase of edema formation), the group treated with 100 mg/kg showed a reduction in edema volume at all tested times. It is known that the second phase is characterized by a high production of prostaglandins that promote edema formation [8]. Our results showed that oleoresin interfered with the production of mediators such as PGE_2_. *C. multijuga* oleoresin and its hexane and chloroform fractions administered at 150 mg/kg were able to reverse the edematogenic effect of carrageenan by at least 50% [19].

In the air pouch model, a thin layer of fibroblasts and macrophages is formed in the intrascapular region closely similar to the synovial region and the administration of carrageenan simulates an inflammatory response in the human joints. Carrageenan activates a Toll-like receptor (Toll 4 type) with the consequent activation of the nuclear transcription factor NF-κB. When activated, this factor results in the production and release of pro-inflammatory cytokines that recruit cells to the site of inflammation, and also results in the production of NO and other inflammatory mediators [33]. Administration of carrageenan induces a response that favors an ideal environment for the collection of many cells and extracellular fluids rich in pro-inflammatory cytokines. These products can be qualified, quantified, and used as evaluative parameters of inflammation [33,34,35]. On this basis, our results showed that *C. reticulata* oleoresin reduced the fluid overflow into the cavity of the air pouch. This effect is probably related to the reduction in vascular permeability through the production and release of inflammatory mediators. The reduction in exudate volume agrees with the reduction in paw edema. It is known that the chemotactic and exudative process is directly related to macrophage activity regarding the production of mediators and the increase in vascular permeability [36]. Therefore, oleoresin may perhaps interfere with macrophage activity by reducing the production and release of inflammatory mediators such as PGE_2_, TNF-α, IL-1β, and NO.

Inducer agents such as carrageenan and lipopolysaccharides (LPS) and pro-inflammatory cytokines such as TNF-α stimulate the expression of induced nitric oxide synthase (iNOS), which regulates the production of NO by macrophages and other cells activated by cytokines [37]. NO has the ability to destroy pathogens by inducing oxidative stress in macrophages or polymorphonuclear cells, also acting on the modulation of cell development and cytokine production, and can act on the endothelium to promote vasodilation, to increase permeability, and produce an exudate [33]. In the present study, oleoresin reduced nitrite levels in the air pouch and consequently vascular permeability, since the volume of exudate was significantly reduced. These results agree with those obtained from the paw edema assay because oleoresin also inhibited the formation of edema in the rat paws. Veiga et al. [13] evaluated oleoresins from some *Copaifera* species and observed the inhibition of nitrergic activity in peritoneal macrophages of LPS-stimulated mice when NO production was inhibited.

Considering the importance of cytokines in the inflammatory process, we investigated the effect of *C. reticulata* oleoresin on TNF-α and IL-1β levels in the exudate formed inside the air pouch. Oleoresin significantly reduced the levels of these cytokines as well as NO levels. Gomes et al. [38] determined the levels of TNF-α in the ascitic fluid extracted from animals with Ehrlich ascites tumor cells and observed a significant reduction after treatment with oleoresin. The present results also agree with Gelmini et al. [39], who determined the production of TNF-α and IL-1β by LPS-stimulated human monocytes pre-treated with a purified fraction of *C.* *langsdorffii* (0.1–100 µM) containing sesquiterpenes (31.74%), diterpenic acids (44.73%), and diterpenes (5.58%).

Macrophages have an important function in the inflammatory response through the production and release of many mediators. When macrophages release TNF-α, arachidonic acid from cell membrane phospholipids is metabolized by cyclooxygenases, yielding prostanoids and PGE_2_ in particular. PGE_2_ acts on vascular permeability and is an important mediator that acts on the development of several inflammatory diseases, on pain induction, and leukocyte recruitment [8]. *C. reticulata* oleoresin reduced the synthesis of PGE_2_ in the air pouch, in agreement with its anti-edematogenic action, inhibiting edema formation in the second phase. The second phase of the paw edema assay is characterized by a high production of prostaglandins which contribute to the maintenance of edema [8]. Thus, a reduction in PGE_2_ seems to be directly related to the decrease in paw edema and exudate in the air pouch.

Veiga et al. [13] evaluated the anti-inflammatory activity of *C. reticulata* (100, 200, and 400 mg/kg) using a zymosan-induced inflammation model in male Swiss mice. Oleoresin inhibited total leukocyte and neutrophil accumulation only at 400 mg/kg but did not inhibit the protein extravasation induced by zymosan. Ghizoni et al. [21] evaluated the action of *C. reticulata* oleoresin on the systemic inflammation of arthritic male Holtzman rats. Severe arthritis in the paw edema model was induced by Freund’s adjuvant (heat-inactivated *Mycobacterium tuberculosis* derived from the H37 Rv human strain) and rats were treated with oleoresin at 580 and 1150 mg/kg. Treatment was only partially effective since it was able to decrease the contralateral paw edema, but not the secondary lesions due to arthritis in the tail and ears. Teixeira et al. [20] investigated the anti-inflammatory activity of *C. reticulata* oleoresin using a model of injury to rat tongues. Oleoresin therapy (200 mg/kg) modulated the inflammatory response by decreasing the chronic inflammatory infiltrate, edema, and specifically the number of macrophages in the injured area.

The present results showed that oleoresin rich in β-Bisabolene extracted from FLONA had a significant anti-inflammatory activity confirmed by the carrageenan-induced inflammation model, reducing vascular permeability and consequently edema formation, and thus reducing cell migration and the production of NO, PGE_2_, IL-1β, and TNF-α. This study supports the anti-inflammatory potential of *C. reticulata* oleoresin, confirming the traditional medicinal use of this natural product by local people in the Amazon region. Further studies are required to evaluate the specific compounds associated with different degrees of anti-inflammatory activity, but the results from this study combined with the recent literature showing a similar activity with different chemical compositions based on volatile compounds may indicate that the Amazon tree Copaiba can be a source for multiple molecules with anti-inflammatory properties [13,16].

## Figures and Tables

**Table 1:** Qualitative and quantitative composition of volatile compounds from *Copaifera reticulata* oleoresin by CG-MS.

t_R_ (min)	Compound	% *
22.55	*β*-elemene	6.90
24.37	*trans*-*α*-bergamotene	12.76
24.55	*cis*-*β*-farnesene	0.41
26.40	*cis*-eudesma-6,11-diene	14.20
26.47	*β*-selinene	8.70
26.75	*α*-selinene	7.03
27.45	*β*-bisabolene	25.15
28.61	*γ*-bisabolene	2.16
29.97	caryophyllene oxide	0.55
31.07	humulene epoxide II	1.21
31.19	8-cedren-13-ol	0.87
31.36	Junenol	2.52
31.54	14-hydroxy-9-*epi*-(E)-caryophyllene	3.27
31.82	5-cedranone	1.01
32.05	*α*-bisabolene epoxide	0.69
32.79	Selin-11-en-4-*α*-ol	4.41
33.50	Caryophylla-4(12),8(13)-dien-5*α*-ol	1.47
33.79	Germacra-4(15),5,10(14)trien-1-*α*-ol	1.21
34.13	*α*-bisabolol	0.67
36.10	*cis*-thujopsenal	0.96
38.45	Eudesm-11-en-4-*α*-,6-*α*-diol	0.58
45.37	Kaurene	1.30

t_R_ = retention time. % = Relative percentage (in relation to total compound found in GC-Ms analysis) * Percentages were calculated only in relation to majority volatile compounds identified.

**Table 2:** Bibliographic references compared to our results.

Compounds *	*C. reticulata* (%) ^1^	*C. reticulata* (%) ^2^	*C. reticulata* (%) ^3^
β-Bisabolene	25.15	0.0050	0.8
Methyl hardwickiiate	-	0.0085	2.3
Germacrene D	-	-	5.0
α-Humulene	-	0.0016	6.0
β-Cariophyllene	-	-	40.9
*Trans*-alpha-bergamotene	12.76	-	-
Cis-eudesma-6,11-diene	14.20	-	-
Aromadendrene	-	0.0120	-
*Trans*-β-cariophyllene	-	0.0900	-

^1^ Samples obtained from FLONA Tapajós, Santarém Pará, Brazil (central Amazon), this study. ^2^ Data from Rio Branco, Acre (western Amazon) [16]. ^3^ Samples obtained from Belém, PA, Brazil (eastern Amazon) [13]. * Percentages were calculated only in relation to majority volatile compounds identified.

## Data Availability

All the raw data collected in this research is fully and completely available upon request to the corresponding author.
